# Hepatic Cytochrome P450 Abundance and Activity in the Developing and Adult Göttingen Minipig: Pivotal Data for PBPK Modeling

**DOI:** 10.3389/fphar.2021.665644

**Published:** 2021-04-15

**Authors:** Laura Buyssens, Laura De Clerck, Wim Schelstraete, Maarten Dhaenens, Dieter Deforce, Miriam Ayuso, Chris Van Ginneken, Steven Van Cruchten

**Affiliations:** ^1^ Comparative Perinatal Development, Department of Veterinary Sciences, Faculty of Pharmaceutical, Biomedical and Veterinary Sciences, University of Antwerp, Wilrijk, Belgium; ^2^ Laboratory of Pharmaceutical Biotechnology, Department of Pharmaceutics, Faculty of Pharmaceutical Sciences, Ghent University, Ghent, Belgium; ^3^ Laboratory of Pharmacology and Toxicology, Department of Pharmacology, Toxicology and Biochemistry, Faculty of Veterinary Medicine, Ghent University, Merelbeke, Belgium

**Keywords:** CYP, protein abundance, ontogeny, Göttingen Minipig, pediatrics, mass spectrometry

## Abstract

The Göttingen Minipig is gaining ground as nonrodent species in safety testing of drugs for pediatric indications. Due to developmental changes in pharmacokinetics and pharmacodynamics, physiologically based pharmacokinetic (PBPK) models are built to better predict drug exposure in children and to aid species selection for nonclinical safety studies. These PBPK models require high quality physiological and ADME data such as protein abundance of drug metabolizing enzymes. These data are available for man and rat, but scarce for the Göttingen Minipig. The aim of this study was to assess hepatic cytochrome P450 (CYP) protein abundance in the developing Göttingen Minipig by using mass spectrometry. In addition, sex-related differences in CYP protein abundance and correlation of CYP enzyme activity with CYP protein abundance were assessed. The following age groups were included: gestational day (GD) 84–86 (*n* = 8), GD 108 (*n* = 6), postnatal day (PND) 1 (*n* = 8), PND 3 (*n* = 8), PND 7 (*n* = 8), PND 28 (*n* = 8) and adult (*n* = 8). Liver microsomes were extracted and protein abundance was compared to that in adult animals. Next, the CYP protein abundance was correlated to CYP enzyme activity in the same biological samples. In general, CYP protein abundance gradually increased during development. However, we observed a stable protein expression over time for CYP4A24 and CYP20A1 and for CYP51A1, a high protein expression during the fetal stages was followed by a decrease during the first month of life and an increase toward adulthood. Sex-related differences were observed for CYP4V2_2a and CYP20A1 at PND 1 with highest expression in females for both isoforms. In the adult samples, sex-related differences were detected for CYP1A1, CYP1A2, CYP2A19, CYP2E1_2, CYP3A22, CYP4V2_2a and CYP4V2_2b with higher values in female compared to male Göttingen Minipigs. The correlation analysis between CYP protein abundance and CYP enzyme activity showed that CYP3A22 protein abundance correlated clearly with the metabolism of midazolam at PND 7. These data are remarkably comparable to human data and provide a valuable step forward in the construction of a neonatal and juvenile Göttingen Minipig PBPK model.

## Introduction

In recent years, the Göttingen Minipig has gained attention in view of pediatric drug development (Downes, 2018). Especially for the youngest age groups (i.e. from birth up to 2 years), the Göttingen Minipig may be a better translational model than rodents due to their comparable body size and organ development but also for their similar drug metabolism (Yoshimatsu et al., 2016; Van Peer et al., 2017; Downes, 2018). In order to compare the juvenile Göttingen Minipig with the human pediatric population, efforts are ongoing to further characterize this animal model and build a physiologically based pharmacokinetic (PBPK) model. PBPK modeling requires three different sources of information namely i) systemic data based upon the population of interest (e.g. organ weight, blood flow rate, amount of microsomal protein per gram of liver (MPPGL), glomerular filtration rate, ontogeny of drug metabolizing enzymes and transporters (DMET)) ii) drug data (e.g. physicochemical parameters, drug solubility, tissue partitioning, plasma protein binding, drug-drug interactions), and iii) trial design parameters (e.g. dose and dose regimen, route of administration, population size and demographics) (Jamei et al., 2009; Zhuang and Lu, 2016). The integration of these various parameters results in a mathematical model that provides a bottom-up approach to predict drug exposure (Zhuang and Lu, 2016). To validate the PBPK model, *in vivo* data are compared to the generated simulations and this leads to a feedback loop that allows for constant refinement of the model (Parrott et al., 2011; Heikkinen et al., 2015). This mechanistic approach has received increased attention, especially for the pediatric population, as it may achieve more accurate dose predictions compared to the traditional methods (e.g. allometric scaling) and consequently results in a better dose setting in this population (Barrett et al., 2012). Age-dependent developmental changes in physiology parameters (e.g. organ size and maturation, plasma protein binding and ontogeny of DMETs) can be specified in the pediatric PBPK model and as such considers important factors that can cause differences in exposure between adults and children of different ages (Duan et al., 2016). Due to the nonlinear changes of these developmental changes, the allometric scaling approach (which estimates the maturation of physiological processes solely based upon body size (Johnson, 2008)) is a rather inappropriate method to scale drug-doses from the adult to the youngest pediatric population (Johnson, 2008). This can be outweighed by PBPK models.

With regard to the Göttingen Minipig, a preliminary PBPK model for the adult population has already been made (Suenderhauf and Parrott, 2013; Suenderhauf et al., 2014) and the first steps have been taken to create a model for the juvenile population (Van Peer et al., 2016). Morphometric organ data and activity data of a limited group of hepatic Phase I (cytochrome P450 (CYP) 3A enzymes) and Phase II (UGT enzymes) drug metabolizing enzymes are already available in juvenile groups (Van Peer et al., 2016; Van Peer et al., 2017). However, data on the biotransformation capacity and other ADME properties in the neonatal and juvenile Göttingen Minipig remain scarce and hamper the development of a reliable model (Van Peer et al., 2017). Thus, further characterization is essential to expand our knowledge in view of the construction of a neonatal and juvenile Göttingen Minipig PBPK model.

This paper focuses on Phase I drug metabolism and more specifically on CYP protein abundance in the liver of the Göttingen Minipig. CYP enzymes are one of the most important Phase I drug metabolizing enzymes, as they are responsible for the biotransformation of 70–80% of drugs in clinical use (Zanger and Schwab, 2013). Oxidation, reduction and hydrolysis reactions of substrates will form more hydrophilic metabolites and this facilitates the biliary and renal excretion or further metabolization through Phase II enzymes (Guengerich, 2008). High homology to the human Phase I drug metabolizing CYP family is described in adult minipigs (63–84% amino acid identity) (Anzenbacher et al., 1998; Soucek et al., 2001; Puccinelli et al., 2011; Heckel et al., 2015; Lignet et al., 2016) and the ontogeny of CYP enzyme activity in the juvenile Göttingen Minipig showed to be comparable to the corresponding age groups in human (Van Peer et al., 2017). However, CYP protein abundance still has to be examined. Determination of the protein abundance levels is critical for the refinement of the neonatal and juvenile Göttingen Minipig PBPK model. In the past, mRNA levels were used as a surrogate for the protein levels of DMETs and showed to not always correlate well (Vogel and Marcotte, 2012; Heikkinen et al., 2015; Ladumor et al., 2019). Hence, proper protein quantitation is necessary. Within this regard, the development of liquid chromatography-mass spectrometry (LC-MS) based quantitative proteomics has increased the expectations for a solid progression in the field as it is considered to be more precise and reliable than other protein quantitation techniques (e.g. Western blot, ELISA) (Ladumor et al., 2019). In our research, a liquid chromatography-tandem mass spectrometry (LC-MS/MS) method (Millecam et al., 2018) was used to detect peptides unique to the various CYP isoforms, based on the retrieved signal intensities of these peptides, protein abundance was determined.

The main goal of this study was to assess the ontogeny of hepatic CYP protein abundance in the Göttingen Minipig with LC-MS/MS. The CYP protein abundance was measured in liver microsomes of both male and female minipigs, with age groups ranging from the late fetal stage to postnatal day (PND) 28. Adults were included for reference. Second, sex-related differences affecting CYP protein abundance were investigated. Third, the ontogeny profiles of CYP enzyme activity and CYP protein abundance were compared to examine the correlation between both parameters.

## Materials and Methods

### Reagents

Sodium pentobarbital 20% (Kela NV, Hoogstraten, Belgium) was used for anesthesia of the animals. A 0.5 M potassium phosphate (K_3_PO_4_) buffer was obtained from Corning Incorporated (NY, United States). Halt™ Protease Inhibitor Single-Use Cocktail (78430) and Pierce® BCA Protein Assay kit with bovine serum albumin (23225) were purchased from Thermo Fisher Scientific (MA, United States). TEABC, DTT, MMTS, CaCl_2_ and DMSO were obtained from Sigma Aldrich (St. Louis, MO, United States). UPLC-water and ACN were purchased from Biosolve (Valkenswaard, Netherlands). Trypsin was obtained from Promega (Madison, WI, United States). Hi3 *E. coli* was purchased from Waters (Zellik, Belgium). Beta-galactosidase was obtained from Sciex (Framingham, MA, United States).

### Animals and Tissue

The protocol, use of animals and research in this study was approved by the Ethical Committee of Animal Experimentation from the University of Antwerp (Belgium) (ECD 2012-30) and adhered to the ‘Principles of Laboratory Animal Care’ (NIH publication Nr 85-23, revised in 1985). The animals and resulting liver microsomes that were collected and assayed by Van Peer et al. were used in this study (Van Peer et al., 2017). Euthanasia of the animals, sampling of the liver and isolation of the liver microsomes were conducted and described before (Van Peer et al., 2017). Ten pregnant sows were a kind gift from Ellegaard Göttingen Minipig A/S (Dalmose, Denmark). Janssen Research (Beerse, Belgium) kindly provided liver samples from four adult male Göttingen Minipigs. The following age groups were investigated: gestational day (GD) 84–86 (*n* = 8), GD 108 (*n* = 6), PND 1 (within 24 h after birth) (*n* = 8), PND 3 (*n* = 8), PND 7 (*n* = 8), PND 28 (*n* = 8) and adult (*n* = 8). Both genders were equally represented in each age group except for PND 28 (three males and five females). The fetal age groups (GD 84–86 and GD 108) refer to 75 and 95% of gestation, respectively, as normal gestation length in the minipig is 112–115 days. The evaluation of the fetal age groups is therefore restricted to the third trimester of fetal development. PND 28 is the age at which piglets are usually weaned in a preclinical setting. The first month of life in the Göttingen Minipig is considered to depict the first two years of life in children (Ema/Chmp/Ich/616110/2018, 2020), when important changes in human CYP activity and expression occur. The ontogeny pattern of CYP protein abundance was investigated in all age groups. For the adult group, the female samples became available in a later phase. Consequently, sex-related differences for the developing age groups were determined during a first experiment using male adult samples as reference and the sex-related differences in the adult age group were determined in a second experiment when adult female samples were available. The age of adult male and female animals ranged between 18–24 months and 14–33 months, respectively. Total protein concentration of the liver microsomes was determined by the Pierce® BCA Protein Assay Kit with bovine serum albumin as a standard.

### CYP Protein Abundance: LC-MS/MS Approach

A high definition–data dependent acquisition (HD-DDA) mass spectrometry (MS) set-up was used to determine protein abundance in the liver microsomes. This method consists of a full scan MS followed directly by a MS^2^ analysis of the precursor ions with the highest signal intensity. These resulting MS/MS spectra were annotated in order to find unique peptides. Two different experiments were conducted. First, the CYP protein abundance maturation pattern over time was determined. Liver microsomes of all different age groups were included. Sex-related differences were investigated in all age groups except for the adult animals. In the latter group, only male individuals were included since female samples were only available in a later phase. Second, CYP protein abundance and sex-related differences were investigated in adult male and female Göttingen Minipig liver microsomes. These experiments were performed as described by Millecam et al. (Millecam et al., 2018). In brief, microsomal proteins (20 µg) of each individual pig were reduced, alkylated, and digested using trypsin prior to MS-analysis. Peptides were resuspended in 0.1% formic acid. Four hundred nanograms sample was spiked with 50 fmol beta-galactosidase and 50 fmol Hi3 *E. coli* standards before injection. The peptides were separated using a nanoscale UPLC system (nanoACQUITY UPLC, Waters, Milford, MA, United States) coupled to a Synapt G2-Si mass spectrometer (Waters). The Q-TOF Synapt G2-Si instrument was set-up for HD-DDA analysis, acquiring full scan MS and MS/MS spectra (m/z at 50–5,000) in resolution mode. Data analysis of the raw files obtained from the Synapt G2-Si was performed in Progenesis QI (Nonlinear Dynamics) version 2.3 (Waters). Peptides with charge C1 were discarded. For relative quantitation, data was normalized to all proteins. For absolute quantitation, data was normalized to Hi3 *E. coli* peptides. Peptide identification was performed with Mascot 2.5 by searching a compiled database of reviewed *Sus scrofa* entries (SwissProt), supplemented with unreviewed CYP proteins and fragments of interest, the cRAP database (laboratory proteins and dust/contact proteins1) and sequences of spiked standard proteins. For relative quantitation, the top three peptides were used and only proteins with at least one unique peptide were further considered. For absolute quantitation, proteins were quantified using the top three unique peptides against Hi3 *E. coli* peptides, and only proteins with at least one unique peptide were further considered. Protein data was exported from ProgenesisQI for further statistical analysis. All unique peptides were validated in Mascot and detection of the proteins was performed in Uniprot and NCBI. A distinction was made between the experimental evidence for the existence of the enzyme at the protein or transcript level. The difference is indicated by an underscore followed by number 1 or number 2, respectively. Evidence at the protein level means that a clear identification by mass spectrometry is available whereas evidence at the transcript level means that cDNA, RT-PCR or Northern blot data are present, but existence is not proven.

### CYP Protein Abundance Vs Cytochrome Enzyme Activity

In this study, liver microsomes were used for which CYP enzyme activity was determined and reported before (Van Peer et al., 2017). In this preceding study, liver microsomes were incubated with a cocktail of CYP substrates including phenacetin, tolbutamide, dextromethorphan and midazolam. These compounds are probe substrates for human CYP1A2, CYP2C9, CYP2D6 and CYP3A4, respectively. Thus, CYP enzyme activity maturation over time was investigated in the same biological samples used in the current work. We therefore performed a correlation study between the hepatic CYP enzyme activity and CYP protein abundance data originating from the same individuals.

### Statistical Analyses

Normality and homogeneity of variances were tested by the Shapiro-Wilk and Levene’s test, respectively. A log-transformation was performed to meet the assumptions for parametric testing, if necessary. One-way ANOVA was used to examine age-related differences in CYP protein abundance. Tukey’s honest significance difference *post hoc* test was used to identify differences between groups. A *p*-value smaller than 0.05 was considered statistically significant. The Student’s t-test was used to detect sex-related differences within each age group and for each CYP isoform. If assumptions could not be met, a non-parametric Mann-Whitney *U* test was performed. The Bonferroni correction adjusted the threshold *p*-value to 0.008 for sex-related differences. A parametric Pearson correlation test was used to identify a correlation between CYP protein abundance from this study and the results from our preceding study that assessed CYP enzyme activity (Van Peer et al., 2017) in all age groups together and in each age group separately. A non-parametric Spearman rank correlation test was performed if assumptions for parametric testing could not be met. The Bonferroni correction adjusted the threshold *p*-value to 0.00012 for the multiple correlation analyses. Statistical analyses were performed in JMP® Pro 14 (SAS Institute Inc., North Carolina, United States). Graphs were made in JMP® Pro 14 (SAS Institute Inc., Cary, NC, United States) and Microsoft Excel® 2016 (Microsoft Corporation, Redmond, WA, United States).

## Results

### General Aspects

**FIGURE 1:**
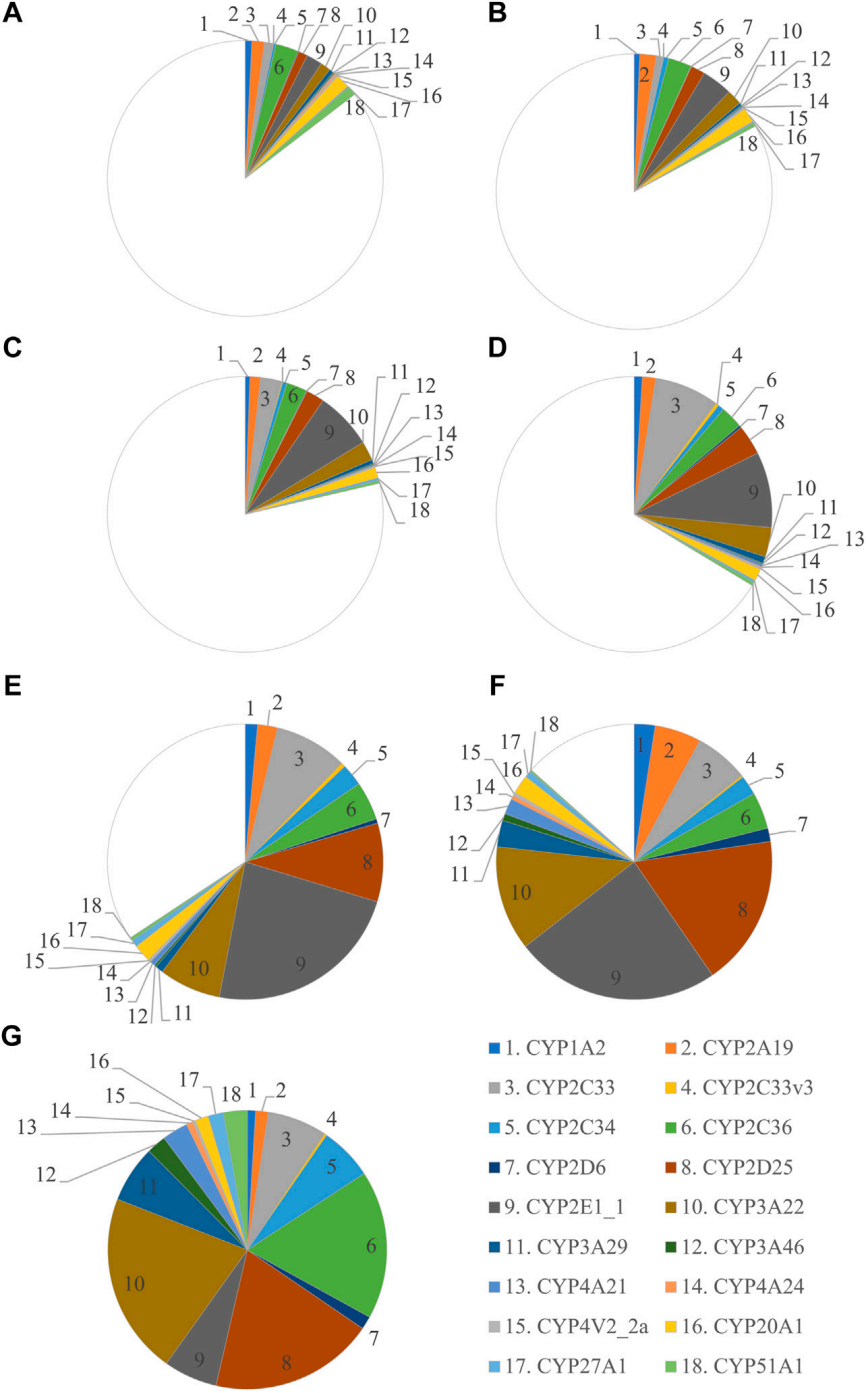
Ontogeny of CYP protein abundance in the developing and adult male Göttingen Minipig. The protein abundance in the male adult samples is considered as the reference and is depicted as a full pie of 100%. The different age groups are represented by GD 84–86 **(A)**, GD 108 **(B)**, PND 1 **(C)**, PND 3 **(D)**, PND 7 **(E)**, PND 28 **(F)** and adult male **(G)**.

**FIGURE 2:**
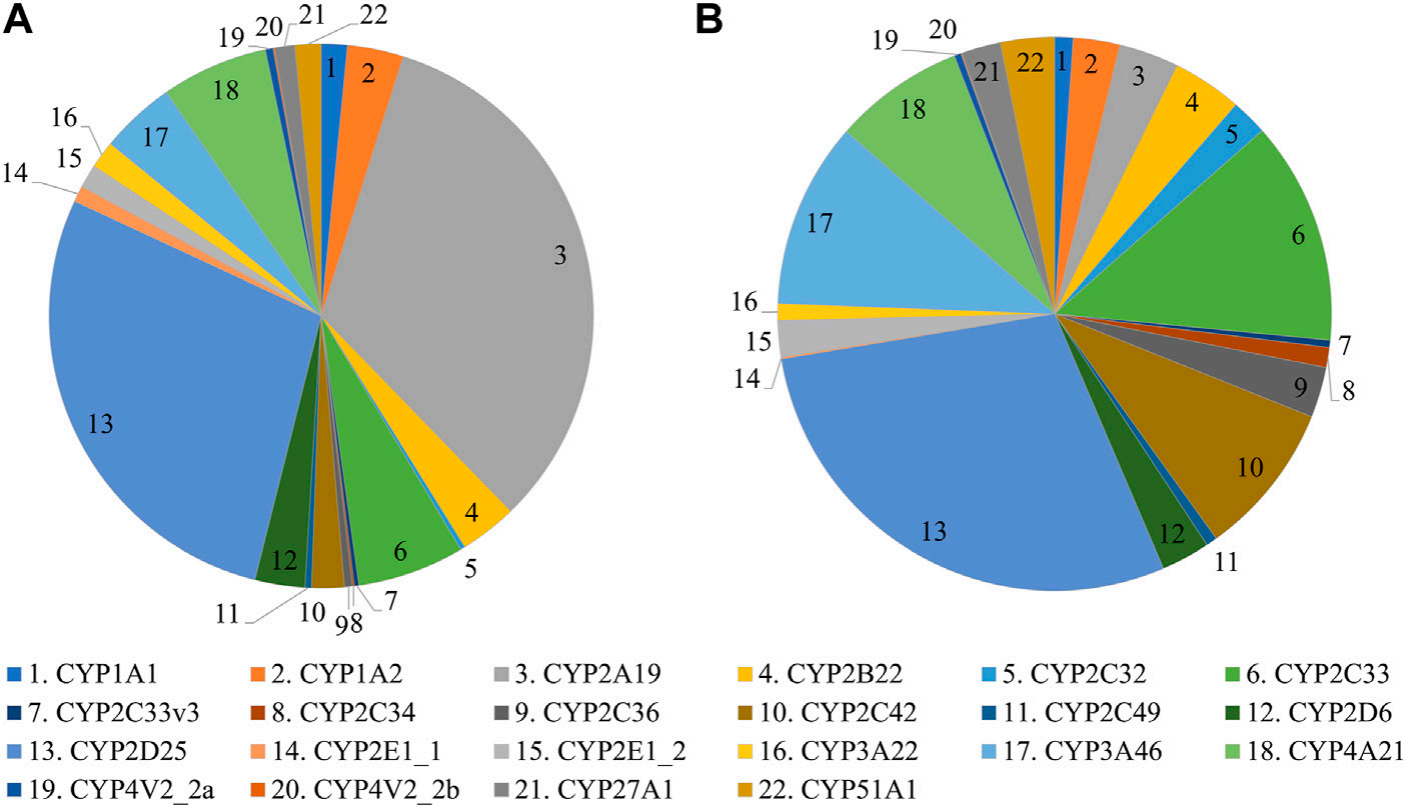
CYP protein abundance profile in adult female **(A)** and male **(B)** Göttingen Minipigs.

In the first experiment, CYP protein abundance was determined in the developing Göttingen Minipig and compared with adult values (Figure 1). A total of 291 proteins were identified in the liver microsomes. Twenty-one CYP enzymes were detected from which 18 had at least one unique peptide. In the second experiment, CYP protein abundance was examined in adult male and female Göttingen Minipig samples (Figure 2). A total of 301 proteins were identified from which 38 belonged to the CYP family. Twenty-two out of 38 CYP enzymes had at least one unique peptide. Fifteen CYP enzymes were detected in both experiments (CYP1A2, CYP2A19, CYP2C33, CYP2C33v3, CYP2C34, CYP2C36, CYP2D6, CYP2D25, CYP2E1_1, CYP3A22, CYP3A46, CYP4A21, CYP4V2_2a, CYP27A1 and CYP51A1). The additional proteins that were found in the first run were CYP3A29, CYP4A24 and CYP20A1; in the second experiment CYP1A1, CYP2B22, CYP2C32, CYP2C42, CYP2C49, CYP2E1_2 and CYP4V2_2b were retrieved.

The most abundant CYP subfamilies that were detected over the course of time in the first experiment were CYP2C, CYP2D, CYP2E and CYP3A (Supplementary Table S1). All these subfamilies could already be detected at the late fetal stages (Figures 1A,B) and showed high values in the different postnatal age groups (Figures 1C–G). In the adult animals of the second experiment, CYP2D25 was the most prominent isoform with 28.8% in males and 28.0% in females (Figure 2) (Supplementary Table S2). Next to this isoform, CYP2C33 (13.0%) was the following most abundant isoform in males and CYP2A19 (32.9%) was the most abundant in females. CYP2A19 only represented 3.5% of total CYP protein abundance in males, CYP2C33 represented 6.4% of total CYP protein abundance in females.

### CYP Protein Abundance Ontogeny Profiles

**FIGURE 3:**
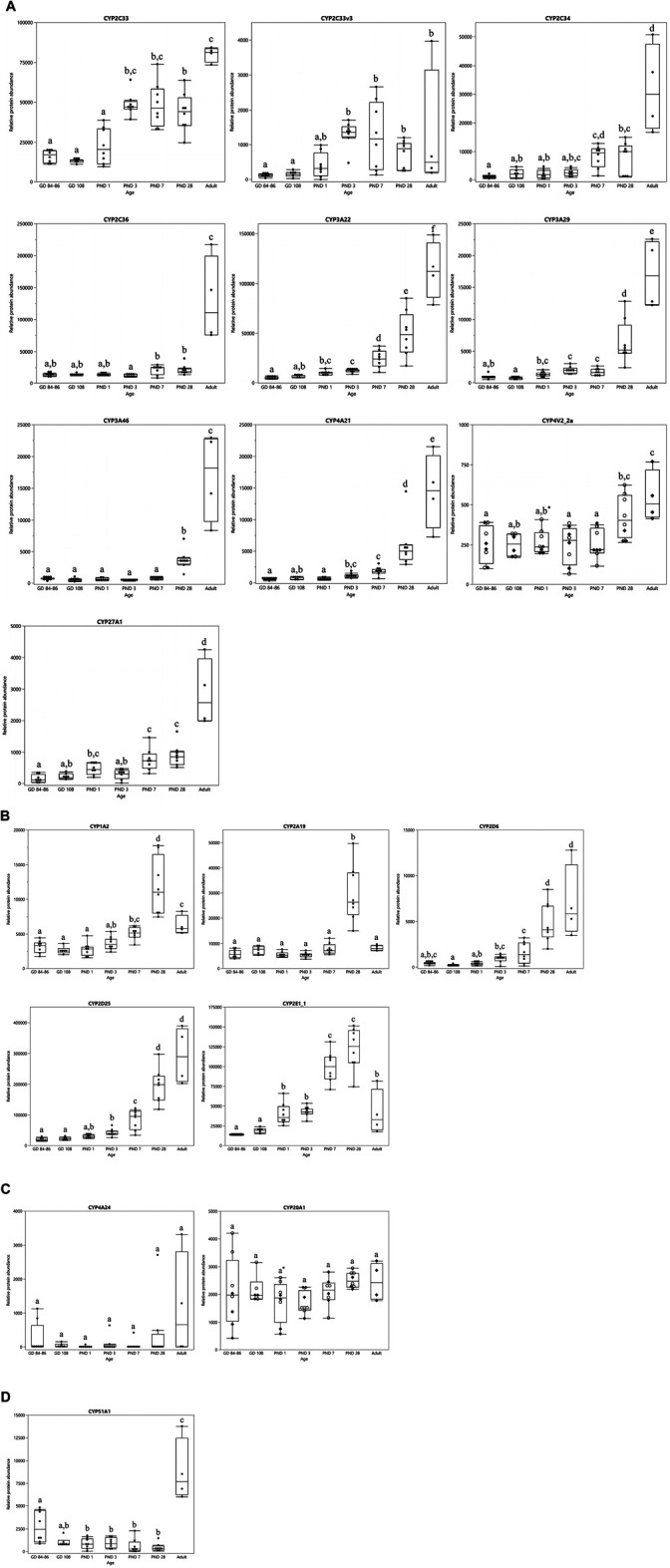
CYP protein abundance maturation profiles over time in the developing and adult male Göttingen Minipig. Different ontogeny patterns were observed: CYP enzymes with a gradual postnatal increase **(A)**, CYP enzymes that reached their maximum value at PND 28 **(B)**, CYP enzymes with a stable expression over time from the fetal stage until the adulthood **(C)** and one atypical CYP isoform with high levels in the fetal stages that dropped during the first month of life and increased again in the adult **(D)**. In case of sex-related differences a distinction is made between female (○) and male (♦) animals, when no sex-related differences are present both sexes are represented by the same symbol (●). The boxplots represent the mean, 25th and 75th quantiles, the upper and lower whiskers indicate the highest and lowest datapoint, respectively, that lies within the 1.5 interquartile range. Datapoints that lie beyond the whiskers are outliers. Statistically significant age-related differences are indicated by characters (*p* < 0.05), statistically significant sex-related differences are indicated by *(*p* < 0.008).

The ontogeny profiles of the individual CYPs showed four different patterns (Figure 3). A gradual increase was observed for CYP2C33, CYP2C33v3, CYP2C34, CYP2C36, CYP3A22, CYP3A29, CYP3A46, CYP4A21, CYP4V2_2a and CYP27A1 (Figure 3A). The highest level of protein abundance for these isoforms was observed at the adult age. CYP1A2, CYP2A19, CYP2D6, CYP2D25 and CYP2E1_1 reached their maximum protein abundance already at PND 28 (Figure 3B). The relative protein abundance of CYP2D6 and CYP2D25 remained unchanged between PND 28 and adulthood, whereas the other isoforms’ abundance dropped. CYP4A24 and CYP20A1 presented a stable protein abundance with no statistically significant differences between the different age groups (Figure 3C). CYP51A1 presented an atypical profile, with high protein abundance in the fetal stages, a drop after birth reaching high abundance again in the adult animals (Figure 3D).

### Sex-Related Differences in CYP Protein Abundance

**FIGURE 4:**
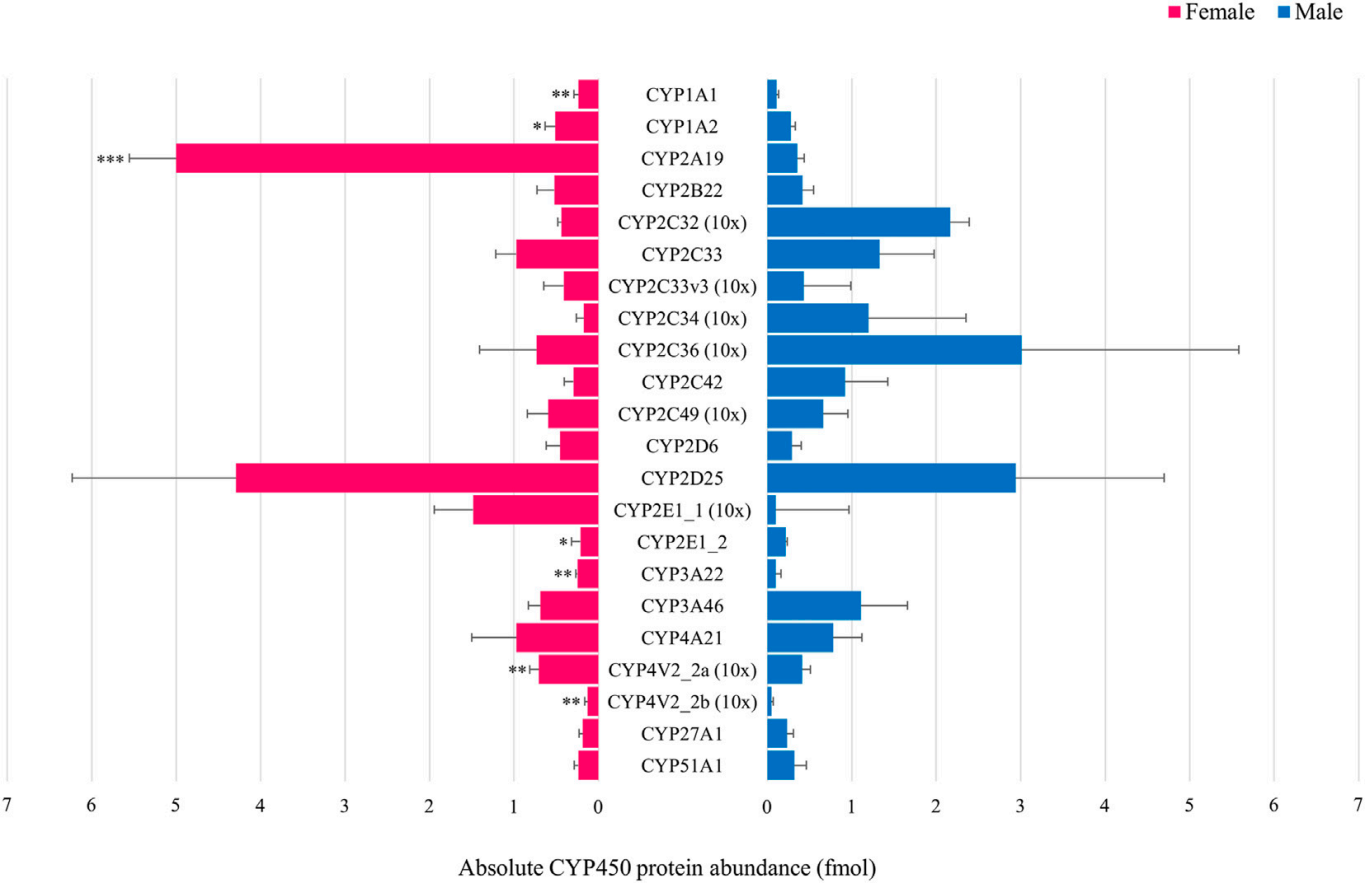
Comparison of the absolute CYP protein abundance in adult female (pink) and male (blue) Göttingen Minipig. Bars represent mean ± S.D. Statistically significant sex-related differences were observed for CYP1A1, CYP1A2, CYP2A19, CYP2E1_2, CYP3A22, CYP4V2_2a and CYP4V2_2b, highest values were observed in the female Göttingen Minipig for these isoforms. For visualization purposes, the CYP2C33, CYP2C33v3, CYP2C34, CYP2C36, CYP2C49, CYP2E1_1, CYP4V2_2a and CYP4V2_2b absolute protein abundance values were magnified 10 times. * (*p* < 0.05), ** (*p* < 0.01), *** (*p* < 0.001).

In the first experiment, only CYP4V2_2a (Figure 3A) and CYP20A1 (Figure 3C) showed statistically significant sex differences at PND 1. CYP protein abundance was highest in female compared to male animals for both isoforms. In the adult age groups of the second experiment, statistically significant sex-related differences were observed for CYP1A1, CYP1A2, CYP2A19, CYP2E1_2, CYP3A22, CYP4V2_2a and CYP4V2_2b with highest values observed in female animals for all isoforms (Figure 4).

### CYP Protein Abundance Vs CYP Enzyme Activity

A Pearson correlation analysis was performed for all age groups together per CYP. Both CYP enzyme activity (determined earlier by the metabolism of phenacetin, midazolam, tolbutamide and dextromethorphan (Van Peer et al., 2017)) and protein abundance showed a similar pattern of postnatal increase for most CYP enzymes, resulting in high correlations between both parameters (Supplementary Table S3). No statistically significant correlation was observed for CYP4A24 and CYP51A1.

Second, Pearson correlation analyses were performed for each CYP per age group (Supplementary Table S4). Only CYP3A22 showed a statistically significant correlation with midazolam metabolism at PND 7 (*p* < 0.00012, *r* = 0.9633). For the youngest age group, GD 84–86, the sample size was too small to include this group in the individual analyses per CYP, as only for 3 samples enzyme activity and protein abundance were assessed.

## Discussion

This study aimed to investigate the hepatic CYP protein abundance in the developing and adult Göttingen Minipig. Since the most important changes in ADME processes occur during the first two years of life in human (Lu and Rosenbaum, 2014), corresponding age groups in Göttingen Minipig were investigated and compared with adult animals. This is the first study assessing changes in protein abundance along development in Göttingen Minipig, although mRNA has been used as a proxy for enzyme activity and protein abundance (Heckel et al., 2015). Since other regulatory processes downstream of transcription, such as posttranslational modifications and protein degradation (Vogel and Marcotte, 2012), determine protein abundance and enzyme activity ontogeny, mRNA abundance was shown to not always be predictive (Vogel and Marcotte, 2012; Prasad et al., 2017; Ladumor et al., 2019). Thus, in order to define drug disposition in the youngest age groups, it is necessary to study all processes (i.e. gene expression, protein abundance and enzyme activity) involved in the ontogeny of DMETs (Prasad et al., 2017).

### General Aspects

In this research, Göttingen Minipig CYP protein abundance was determined in liver microsomes using a LC-MS/MS approach. This technique has become the new standard as immunoaffinity assays (e.g. Western Blot, ELISA etc.) have shown to be not specific enough to quantify different CYP isoforms within the same subfamily (Prasad et al., 2017).

We observed some discrepancies between the two experiments regarding the number of identified CYP isoforms. Fifteen CYP enzymes were detected in both runs (CYP1A2, CYP2A19, CYP2C33, CYP2C33v3, CYP2C34, CYP2C36, CYP2D6, CYP2D25, CYP2E1_1, CYP3A22, CYP3A46, CYP4A21, CYP4V2_2a, CYP27A1 and CYP51A1) which were completed by three (CYP3A29, CYP4A24 and CYP20A1) and seven (CYP1A1, CYP2B22, CYP2C32, CYP2C42, CYP2C49, CYP2E1_2 and CYP4V2_2b) additional isoforms in the first and second experiment, respectively. This disparity can be explained by the fact that both experiments were performed separately at different times. This may cause small differences in the results. In addition, HD-DDA is confined by the limited reproducibility that derives from the stochastic precursor ion selection. Moreover, HD-DDA is not suitable for low abundant precursor ions because they may be never selected for fragmentation. So, the differences between the two experiments are more likely due to technical reasons than to real differences in protein abundance.

CYP2C, CYP2D, CYP2E and CYP3A were found to be the most abundant CYP subfamilies in the different age groups of the first experiment. These results agree with the hepatic CYP protein abundance profile of 12-week-old conventional pigs (hybrid sow x Piétrain boars, 8 male and 8 female animals) (Schelstraete et al., 2019). In the latter, CYP2A was found to be the most abundant in the liver, followed by CYP2D, CYP3A, CYP2E, CYP2C and CYP1A (Schelstraete et al., 2019). In our second experiment, CYP2C, CYP2D and CYP3A subfamilies were the most abundant in the male adult Göttingen Minipig whereas the CYP2A, CYP2C and CYP2D subfamilies were the most abundant in adult females. This accords with previous research (Achour et al., 2011; Millecam et al., 2018), although CYP2A19 had a remarkably lower abundance (3.5%) in males in ours compared to these other studies. CYP2A19 was the most abundant CYP isoform in 2 adult male Suffolk White pigs (34%) (Achour et al., 2011) and was among the most abundant isoforms in 6-month-old male conventional pigs (Large white x Land race, Seghers hybrid) (12%) (Millecam et al., 2018). Based on these differences, CYP2A19 protein abundance could be breed-dependent, but data regarding its ontogeny remain scarce. Nevertheless, CYP2A19 sex-related differences with higher protein expression in female in comparison to male Göttingen Minipig, were in accordance with earlier studies (Rasmussen et al., 2011; Kojima and Degawa, 2016; Millecam et al., 2018). Sex-related differences in CYP abundance will be discussed later.

### CYP Protein Abundance Ontogeny Profiles

In general, a gradual postnatal increase in protein abundance was observed for most CYP isoforms, although with some exceptions, such as CYP4A24 and CYP20A1. These isoforms were stable over time and were already present in the late fetal stages. As CYP4A24 is not solely responsible for drug metabolism but also for physiological functions (e.g. fatty acid metabolism), this may explain this pattern (Okita and Okita, 2001; Lundell, 2002). Moreover, this CYP4A24 maturation pattern is consistent with the human CYP4A11 ontogeny profile which shows a constant gene and protein expression over time (Sadler et al., 2016). CYP20A1, which function is still unknown, shows a fairly constant protein abundance pattern in human, similarly to what we observed in the Göttingen Minipig (Guengerich et al., 2005; Nebert et al., 2013; Sadler et al., 2016). CYP51A1 demonstrated higher protein abundance in the late fetal stages than during the first month of life, which also agrees with previous findings in human (Sadler et al., 2016). CYP1A2, CYP2A19, CYP2D6, CYP2D25 and CYP2E1_1 showed their highest protein abundance at PND 28. This is in accordance with a gene expression and enzyme activity study by Heckel et al. who suggest that the ontogeny of CYP genes is completed at four weeks of age in the Göttingen Minipig (Heckel et al., 2015). Based on these findings, they suggest that 4-week-old piglets give similar pharmacological responses as adult minipigs. However, our results warrant caution since this seems not to be the case for all isoforms. CYP2C33, CYP2C34, CYP2C36, CYP3A22, CYP3A29, CYP3A46, CYP4A21, CYP4V2_2a and CYP27A1 reached their highest level only at the adult age. This illustrates again that gene expression, protein abundance and enzyme activity are not always interchangeable.

Hines et al. (Hines, 2013) recently classified human hepatic DMET ontogeny profiles into three different classes based upon their onset and expression pattern. Class 1 DMETs have their highest expression during the first trimester of gestation (e.g. CYP51A1), Class 2 DMETs show a constant expression from pregnancy until adulthood (e.g. CYP2B6 and CYP20A1) and Class 3 consists of DMETs with a negligible expression during gestation, and even low expression at birth that rises postnatally during maturation (Hines, 2013; Sadler et al., 2016). Class 3 is the largest group within this classification comprising CYP1A2, CYP2A6, CYP2C8, CYP2C9, CYP2D6, CYP2E1, CYP2J2, CYP3A4, CYP4F11, CYP4V2 and CYP27A1 (Stevens et al., 2008; Hines, 2013; Sadler et al., 2016). When comparing their results to our findings, the CYP2A, CYP2C, CYP2D, CYP3A, CYP4V, CYP20A, CYP27A and CYP51A subfamilies belong to the same classes in both species. Thus, we can say that the hepatic CYP protein abundance ontogeny is highly comparable between Göttingen Minipig and human. However, some discrepancies regarding the CYP1A subfamily should be mentioned. In human, CYP1A1 mRNA and protein expression were detected during the first and second trimester of gestation but their expression declined to non-detectable levels toward adulthood (Pasanen et al., 1987; Omiecinski et al., 1990; Murray et al., 1992; Yang et al., 1995; Shimada et al., 1996; Hines and McCarver, 2002). CYP1A1 is thus considered to be a player in fetal xenobiotic metabolism (Hines and McCarver, 2002). In our study, conversely, CYP1A1 was only detected in the adult Göttingen Minipigs of the second experiment. It is debatable whether this is caused by the technical variability of both LC-MS/MS experiments, or whether it is a species-specific characteristic. In the past, CYP1A1 mRNA was detected in both fetal and adult porcine liver samples whereas CYP1A1 protein was solely retrieved in adult animals (Rasmussen et al., 2016). On the other hand, CYP1A2 mRNA, protein and enzyme activity were detected only after birth in human infants (Mäenpää et al., 1993; Hakkola et al., 1994; Yang et al., 1995; Shimada et al., 1996; Sonnier and Cresteil, 1998). For this isoform, a partly comparable pattern is observed in the Göttingen Minipig: CYP1A2 protein abundance only starts to increase postnatally from PND 3 onwards, which accords to the human situation. Nevertheless, the protein is already observed in the fetal age groups which is not the case in human. Thus, using the (mini)pig as a translational model for the human CYP1A subfamily should be considered carefully.

Next to the human population, large similarities in CYP protein abundance were also observed between the Göttingen Minipig and the conventional pig. Millecam et al. investigated CYP protein abundance in conventional pigs of 2 days, 4 weeks, 8 weeks and 6–7 months of age (Millecam et al., 2018). Since the investigated age groups are not entirely the same compared to our study, caution is needed when drawing conclusions, especially for the youngest age groups. CYP1A2, CYP2C34, CYP2C36, CYP2C49, CYP2D6, CYP3A22, CYP3A29, CYP3A46, CYP4A21, CYP4V2_2a and CYP51A1 abundance increased in a similar way in the conventional pig and the Göttingen Minipig. On the other hand, CYP2E1_1 and CYP20A1 appeared to have a different ontogeny profile in the conventional pig and the Göttingen Minipig. CYP2E1_1 had a stable protein abundance profile over time in the conventional pig whereas in the Göttingen Minipig, a gradual postnatal increase was observed. This difference may be due to different environmental factors (e.g. diet, (un)controlled housing conditions etc.), and to breed-related genetic variations (Burkina et al., 2019). In humans, however, the CYP2E1 ontogeny pattern shows a gradual postnatal increase, specifically from the first month of life until 1 year of age (Vieira et al., 1996; Johnsrud et al., 2003). Thus, the Göttingen Minipig may be a better translational model for human CYP2E1 than the conventional pig. In addition, Göttingen Minipig CYP2E1 substrate specificity was already shown to be similar to human CYP2E1 (Baranová et al., 2005). With regard to CYP20A1, protein ontogeny remains stable from the late fetal stage until adulthood in the Göttingen Minipig whereas in the conventional pig, a significant difference is observed between the youngest age groups and the animals of 6–7 months of age (Millecam et al., 2018). Since CYP20A1 is still considered as an orphan CYP, data is limited, also in human (Guengerich et al., 2005). Therefore, we cannot conclude at present which breed represents better the human situation for this enzyme.

Besides the ontogeny profiles, our study provides additional data regarding the detection of CYP proteins in fetal porcine liver samples. Earlier immunochemical assays were not able to detect CYP1A, CYP2A, CYP2E and CYP3A subfamilies in porcine fetuses (Rasmussen et al., 2016). The proteomic approach that was used in this study proved to have better sensitivity than immunochemistry and provided evidence that these subfamilies are already present by the third trimester of gestation.

### Sex-Related Differences in CYP Protein Abundance in the Developing Göttingen Minipig

For the majority of the detected CYP isoforms, no sex-related differences were observed during the first month of life. This agrees with previous findings in juvenile Göttingen Minipigs and conventional pigs regarding CYP enzyme activity and protein abundance (Van Peer et al., 2017; Millecam et al., 2018; Schelstraete et al., 2019). The fact that 28-day-old Göttingen Minipigs do not present sex-related differences and adult Göttingen Minipigs do, can be explained by the changed expression of growth and sex hormones that occurs during puberty (Kennedy, 2008). The effect of sex hormones was for example illustrated by suppressed CYP1A, 2A and 2E1 mRNA expression in entire, i.e. non-castrated, male pigs compared to surgically and immunologically castrated male pigs from Meishan (Kojima et al., 2008), Yorkshire x Landrace (Brunius et al., 2012) and Large White x Landrace x Duroc (Kubešová et al., 2019) strains. In entire Bama miniature male pigs, a decrease of CYP2A19 and CYP2E1 mRNA expression was observed after 6 months due to sexual maturity (Wang et al., 2015). However, suppressed CYP mRNA expression did not always result in decreased CYP protein and enzyme activity (e.g. CYP2E1) (Brunius et al., 2012). In this case, posttranslational modifications may be involved as a regulatory mechanism (Brunius et al., 2012), confirming again that all parameters (i.e. gene expression, protein expression and enzyme activity) should be taken into account. Comparative data regarding CYP expression in entire and castrated male Göttingen Minipigs are not available yet, but a similar outcome may be expected.

Nevertheless, sex-related differences were present for CYP4V2_2a and CYP20A1 at PND 1, with females having higher protein abundance than males. However, these isoforms are not relevant for drug metabolism and their importance may lie elsewhere (Guengerich et al., 2005; Nakano et al., 2009; Nebert et al., 2013; Sadler et al., 2016).

In our preceding study, a significantly higher CYP activity was found in female Göttingen Minipigs for tolbutamide metabolism (human CYP2C9 substrate) at PND 1 and phenacetin metabolism (human CYP1A2 substrate) at PND 28 and adults (Van Peer et al., 2017). These observations were not confirmed in the current study, except for the phenacetin metabolism in adults which can be linked to the higher CYP1A protein abundance in female adult Göttingen Minipigs (see next section). Regarding the other substrates (midazolam and dextromethorphan, human CYP 3A4 and CYP2D6 substrates, respectively) no sex-related differences were previously determined (Van Peer et al., 2017). These observations are partly confirmed in our study since CYP2D and CYP3A isoforms did not show sex-related differences during maturation. However, a discrepancy regarding sex-related differences in CYP3A activity and protein abundance is noticed for the adult age (see next section).

As described above, the sex-related differences that were observed during development (CYP4V2_2a and CYP20A1 at PND 1), were detected in CYP isoforms that are not involved in drug metabolism (Guengerich et al., 2005; Nakano et al., 2009; Nebert et al., 2013; Sadler et al., 2016). Nevertheless, it may be interesting to look further into these observations as sex-related differences were observed in brain and GI metabolism before (Vázquez-Gómez et al., 2016; Kingsbury and Bilbo, 2019) and were linked to better resilience in female human and porcine neonates (Baxter et al., 2012; Muns et al., 2016). Since no sex-related differences were observed at the transcriptional level during the first four weeks of life in Göttingen Minipig liver (Heckel et al., 2015), varying posttranscriptional modifications and protein half-life between sexes, and error/noise in these kind of high throughput experiments (Greenbaum et al., 2003) have to be considered to explain these observations. In addition, the effect of birth should also be examined as a possible factor because the only sex-related differences before puberty were observed at PND 1. Interestingly, sex-related differences regarding hormone levels have been described in neonates before (Alur, 2019; Kingsbury and Bilbo, 2019); this supports the idea to look further into the influence of this event. Hence, it is unclear which impact these results may have, especially since these differences were observed at a single time point. In summary, further research is required to comprehend these findings but their relevance is out of the scope of drug development.

### Sex-Related Differences in CYP Protein Abundance in the Adult Göttingen Minipig

In adult Göttingen Minipig, CYP1A1, CYP1A2, CYP2A19, CYP2E1_2, CYP3A22, CYP4V2_2a and CYP4V2_2b protein abundance was significantly higher in females compared to males. This is in accordance with previous findings in the Göttingen Minipig (Skaanild and Friis, 1999; Gillberg et al., 2006; Skaanild, 2006), but also in the Yucatan minipig (Bogaards et al., 2000) and the conventional pig (Millecam et al., 2018). In the latter, however, sex-related differences were also observed for CYP2C36 which was not the case in our study.

In human, CYP enzyme activity studies showed higher clearance in males for CYP1A2- (Caucasian population only) (Shimada et al., 1994) and CYP2E1-associated substrates (Sato et al., 1975; Chen et al., 2002; Franconi et al., 2007), whereas CYP3A4- (Austin et al., 1980; Kahan et al., 1986; Hulst et al., 1994; Dilger et al., 1999; Krecic-Shepard et al., 2000) and CYP2B6-associated (Lamba et al., 2003) clearance were higher in females for various substrates, although conflicting data have been reported and interindividual differences may prevail (Yang et al., 2013; Tracy et al., 2016). Conflicting results were also obtained for CYP2C9 (Houghton et al., 1975; Rugstad et al., 1986; Karim et al., 1997), CYP2C19 (Richardson et al., 1985; Rugstad et al., 1986; Hooper and Qing, 1990; Laine et al., 2000) and CYP2D6 (Walle et al., 1989; Pritchard et al., 1992; Tamminga et al., 1999; Labbé et al., 2000; Hägg et al., 2001; McCune et al., 2001; Aichhorn et al., 2007) depending on the substrate. Moreover, CYP2C19 and CYP2D6 are highly polymorphic genes, which makes it even more challenging to draw conclusions (Goldstein, 2001; Cascorbi, 2003). CYP gene expression studies on the other hand showed significant higher levels for females in comparison to males for among others CYP2A6, CYP2A7, CYP2A13, CYP2B6, CYP3A4, CYP3A5, CYP3A43, CYP27A1 and CYP51A1 (Yang et al., 2012). Thus, in view of our results, the CYP2A and CYP3A subfamilies are comparable between human and Göttingen Minipig. As the CYP3A subfamily is clinically the most important subfamily for drug metabolism in human (Williams et al., 2002), this is an additional asset for the Göttingen Minipig as a translational model for the human population. For the other subfamilies, opposite sex-related differences between both species (CYP1A and CYP2E) or absent differences in Göttingen Minipig (CYP27A1 and CYP51A1) were observed. These differences should be considered when looking for the most appropriate animal model in nonclinical drug development.

### CYP Protein Abundance Vs CYP Enzyme Activity

Göttingen Minipig CYP enzyme activity that was previously determined and described (Van Peer et al., 2017) was correlated to the CYP protein abundance from this study. Since the same biological samples were used for both experiments, a one-on-one correlation between both parameters was possible. We first performed a correlation study for all age groups together. Since most CYPs showed a postnatal increase in enzyme activity and protein abundance, high correlations were observed when comparing both parameters.

Second, correlation within each individual age group and per CYP isoform was assessed. Only CYP3A22 showed a statistically significant correlation between its protein abundance and the metabolism of midazolam at PND 7. This agrees with previous studies in minipig (Bian et al., 2015; Lignet et al., 2016; Van Peer et al., 2017) and pig (Hu, 2015; Millecam et al., 2018; Schelstraete et al., 2019). Regarding the other substrates, the results are striking since several studies have already suggested a link between CYP1A, CYP2C and CYP2D and the metabolism of phenacetin (Hu, 2015), tolbutamide (Anzenbacher et al., 1998; Soucek et al., 2001; Skaanild and Friis, 2008; Hu, 2015; Millecam et al., 2018; Schelstraete et al., 2019) and dextromethorphan (Thörn et al., 2011; Schelstraete et al., 2019), respectively, in various minipig and pig strains. However, in the majority of those studies, no correlation analysis was conducted, but rather the ability to metabolize the substrate. Hence, no direct link between a specific CYP isoform and the substrates could be made. A word of caution is thus required when performing and interpreting such correlation and activity analyses. Moreover, these findings illustrate the importance of further characterizing the Göttingen Minipig CYP enzymes for example by the development of recombinant enzymes. This is the only way to elucidate substrate specificity for the different CYP isoforms.

### Future Potential for PBPK Modeling

The CYP protein abundance data of this study are a valuable step forward in our comprehension of CYP ontogeny in the fetal, neonatal and juvenile Göttingen Minipig. However, in order to extrapolate data from liver microsomes to the entire organ, scaling factors such as the MPPGL are required (Jones and Rowland-Yeo, 2013). Only then the total amount of protein, enzyme activity, etc. can be calculated and implemented into the PBPK model. At this moment, MPPGL data are not yet available for the Göttingen Minipig. Once these data are obtained and the requirements are met, our CYP protein abundance data will be highly valuable for the development of a neonatal and juvenile Göttingen Minipig PBPK model.

## Conclusion

This study was the first to investigate CYP protein abundance in the developing and adult Göttingen Minipig by a proteomic approach. In general, CYP protein abundance was highest in adult animals. However, several CYP proteins were already detected in the late fetal age groups and a significant postnatal increase was present during the first month of life. Sex-related differences were observed in the developing and adult Göttingen Minipig. These data are remarkably comparable to human data and provide a valuable step forward in the construction of a neonatal and juvenile Göttingen Minipig PBPK model.

## Data Availability

The mass spectrometry proteomics data have been deposited to the ProteomeXchange Consortium via the PRIDE (Perez-Riverol et al., 2019) partner repository with the dataset identifier PXD024158.

## Abbreviations

ACN, Acetonitrile; ADME, Administration, distribution, metabolism and excretion; CaCl_2_, Calcium chloride; cDNA, Complement DNA; CYP, Cytochrome P450; DMET, Drug metabolizing enzymes and transporters; DMSO, Dimethyl sulfoxide; DTT, Dithiothreitol; ELISA, Enzyme-linked immunosorbent assay; ESI, Electrospray ionization; GD, Gestational day; GI, Gastro-intestinal; HD-DDA, High definition-data dependent analysis; K_3_PO_4_, Potassium phosphate; LC-MS, Liquid chromatography-mass spectrometry; LC-MS/MS, Liquid chromatography tandem mass spectrometry; MMTS, Methyl methanethiosulfonate; MPPGL, Microsomal protein per Gram of liver; MS, Mass spectrometry; PBPK, Physiologically based pharmacokinetic modeling; PD, Pharmacodynamics; PK, Pharmacokinetics; PND, Postnatal day; Q-TOF, Quadrupole-time of flight; RT-PCR, Real-time–polymerase chain reaction; TEABC, Triethylammonium bicarbonate buffer; UGT, Uridine 5′-diphospso-glucuronosyltransferase; UPLC, Ultra-performance liquid chromatography
