# Supplementary material for: Hepatic Cytochrome P450 Abundance and Activity in the Developing and Adult Göttingen Minipig: Pivotal Data for PBPK Modeling
Source: Front Pharmacol. 2021 Apr 15;12:665644. doi: 10.3389/fphar.2021.665644 (PMC8082684; doi:10.3389/fphar.2021.665644)
Supplement: Supplementary file 1 [file datasheet1.docx]

***Supplementary material***

**Supplementary Table S1**: Distribution of CYP isoforms (%) in liver microsomes of the developing Göttingen Minipig relative to adult male Göttingen Minipigs; complementary to Figure 1.

| CYP | GD 84 – 86 | GD 108 | PND 1 | PND 3 | PND 7 | PND 28 | Adult male |
| --- | --- | --- | --- | --- | --- | --- | --- |
| 1. CYP1A2 | 0.78 | 0.67 | 0.53 | 0.97 | 1.42 | 2.46 | 0.95 |
| 2. CYP2A19 | 1.47 | 1.91 | 1.24 | 1.52 | 2.32 | 5.45 | 1.46 |
| 3. CYP2C33 | 1.04 | 0.82 | 2.53 | 7.61 | 8.59 | 6.32 | 6.93 |
| 4. CYP2C33v3 | 0.03 | 0.04 | 0.11 | 0.38 | 0.47 | 0.22 | 0.28 |
| 5. CYP2C34 | 0.25 | 0.62 | 0.49 | 0.71 | 2.63 | 2.32 | 6.17 |
| 6. CYP2C36 | 2.69 | 2.72 | 2.42 | 2.67 | 4.53 | 4.28 | 17.23 |
| 7. CYP2D6 | 0.10 | 0.05 | 0.09 | 0.28 | 0.51 | 1.58 | 1.52 |
| 8. CYP2D25 | 1.08 | 1.70 | 2.15 | 3.53 | 9.22 | 17.74 | 19.07 |
| 9. CYP2E1_2 | 1.86 | 3.57 | 6.73 | 8.87 | 23.33 | 24.10 | 6.25 |
| 10. CYP3A22 | 1.20 | 1.79 | 2.24 | 3.44 | 7.26 | 12.29 | 21.06 |
| 11. CYP3A29 | 0.47 | 0.31 | 0.46 | 0.78 | 0.93 | 3.09 | 6.60 |
| 12. CYP3A46 | 0.16 | 0.12 | 0.14 | 0.15 | 0.25 | 0.83 | 2.29 |
| 13. CYP4A21 | 0.12 | 0.19 | 0.14 | 0.30 | 0.59 | 1.85 | 3.00 |
| 14. CYP4A24 | 0.22 | 0.04 | 0.00 | 0.09 | 0.09 | 0.49 | 0.75 |
| 15. CYP4V2_2a | 0.19 | 0.20 | 0.19 | 0.21 | 0.25 | 0.37 | 0.34 |
| 16. CYP20A1 | 1.67 | 1.75 | 1.26 | 1.45 | 2.10 | 2.22 | 1.53 |
| 17. CYP27A1 | 0.11 | 0.18 | 0.33 | 0.26 | 0.80 | 0.78 | 1.79 |
| 18. CYP51A1 | 1.12 | 0.43 | 0.32 | 0.42 | 0.39 | 0.20 | 2.76 |
| TOTAL | 15 | 17.11 | 21 | 34 | 66 | 87 | 100.00 |

**Supplementary Table S2:** Distribution of CYP isoforms (%) in female and male adult Göttingen Minipig; complementary to Figure 2.

| CYP | Female | Male |
| --- | --- | --- |
| 1. CYP1A1 | 1.54 | 1.07 |
| 2. CYP1A2 | 3.34 | 2.73 |
| 3. CYP2A19 | 32.87 | 3.49 |
| 4. CYP2B22 | 3.41 | 4.07 |
| 5. CYP2C32 | 0.29 | 2.12 |
| 6. CYP2C33 | 6.35 | 13.03 |
| 7. CYP2C33v3 | 0.27 | 0.42 |
| 8. CYP2C34 | 0.11 | 1.17 |
| 9. CYP2C36 | 0.48 | 2.95 |
| 10. CYP2C42 | 1.93 | 9.01 |
| 11. CYP2C49 | 0.39 | 0.65 |
| 12. CYP2D6 | 2.96 | 2.85 |
| 13. CYP2D25 | 28.00 | 28.82 |
| 14. CYP2E1_1 | 0.97 | 0.10 |
| 15. CYP2E1_2 | 1.39 | 2.13 |
| 16. CYP3A22 | 1.62 | 0.98 |
| 17. CYP3A46 | 4.47 | 10.86 |
| 18. CYP4A21 | 6.33 | 7.65 |
| 19. CYP4V2_2a | 0.46 | 0.41 |
| 20. CYP4V2_2b | 0.08 | 0.05 |
| 21. CYP27A1 | 1.21 | 2.30 |
| 22. CYP51A1 | 1.55 | 3.15 |
| TOTAL % CYP | 100.00 | 100.00 |

**Supplementary Table S3**: Pearson correlation analysis between CYP enzyme activity and protein abundance detected in liver microsomes of the developing and adult Göttingen Minipig. All age groups of the first experiment (GD 84-86, GD 108, PND 1, PND 3, PND 7, PND 28 and male adults) were included. CYP enzyme activity was determined before by investigating the capacity to metabolize 4 known human substrates (i.e. phenacetin, midazolam, tolbutamide and dextromethorphan) in the same liver microsomes as used in this study. The metabolite formation velocities (pmol/min/mg microsomal protein) of these substrates were used for the current correlation analyses.

|  | Phenacetin  (human CYP1A2) | | Midazolam  (human CYP3A4) | | Tolbutamide  (human CYP2C9) | | Dextromethorphan  (human CYP2D6) | |
| --- | --- | --- | --- | --- | --- | --- | --- | --- |
|  | Pearson correlation coefficient | p-value | Pearson correlation coefficient | p-value | Pearson correlation coefficient | p-value | Pearson correlation coefficient | p-value |
| CYP1A2 | 0.818 | <0.0001 | 0.7741 | <0.0001 | 0.6994 | <0.0001 | 0.6567 | <0.0001 |
| CYP2A19 | 0.6152 | <0.0001 | 0.5515 | <0.0001 | 0.4673 | 0.0012 | 0.476 | 0.0009 |
| CYP2C33 | 0.7778 | <0.0001 | 0.8254 | <0.0001 | 0.8501 | <0.0001 | 0.7782 | <0.0001 |
| CYP2C33v3 | 0.5535 | <0.0001 | 0.555 | <0.0001 | 0.4835 | 0.0009 | 0.6325 | <0.0001 |
| CYP2C34 (Fragment) | 0.5756 | <0.0001 | 0.6778 | <0.0001 | 0.7107 | <0.0001 | 0.6083 | <0.0001 |
| CYP2C36 | 0.453 | 0.0018 | 0.6022 | <0.0001 | 0.6946 | <0.0001 | 0.4723 | 0.0011 |
| CYP2D6 (Fragment) | 0.8117 | <0.0001 | 0.8445 | <0.0001 | 0.8511 | <0.0001 | 0.7894 | <0.0001 |
| CYP2D25 | 0.8918 | <0.0001 | 0.9391 | <0.0001 | 0.9115 | <0.0001 | 0.8635 | <0.0001 |
| CYP2E1_1 | 0.814 | <0.0001 | 0.7229 | <0.0001 | 0.6001 | <0.0001 | 0.763 | <0.0001 |
| CYP3A | 0.7841 | <0.0001 | 0.8582 | <0.0001 | 0.8616 | <0.0001 | 0.7559 | <0.0001 |
| CYP3A22 | 0.842 | <0.0001 | 0.9322 | <0.0001 | 0.9147 | <0.0001 | 0.8174 | <0.0001 |
| CYP3A46 | 0.6653 | <0.0001 | 0.7538 | <0.0001 | 0.7702 | <0.0001 | 0.5817 | <0.0001 |
| CYP4A | 0.3135 | 0.1664 | 0.37 | 0.0987 | 0.3107 | 0.1704 | 0.2432 | 0.2881 |
| CYP4A21 | 0.7695 | <0.0001 | 0.8724 | <0.0001 | 0.8509 | <0.0001 | 0.7486 | <0.0001 |
| CYP4V2_2a (Fragment) | 0.3829 | 0.0094 | 0.3789 | 0.0103 | 0.4259 | 0.0035 | 0.2944 | 0.0496 |
| CYP20A1 | 0.341 | 0.0219 | 0.3379 | 0.0232 | 0.3812 | 0.0098 | 0.3275 | 0.0281 |
| CYP27A1 (Fragment) | 0.625 | <0.0001 | 0.6985 | <0.0001 | 0.6336 | <0.0001 | 0.6819 | <0.0001 |
| CYP51A1 | -0.1734 | 0.2545 | -0.0977 | 0.5232 | 0.0008 | 0.9957 | -0.2204 | 0.1457 |

**Supplementary Table S4.1**: Pearson correlation analysis between CYP enzyme activity and protein abundance in liver microsomes originating from Göttingen Minipigs at GD 108. The analysis was performed for each CYP individually. The Bonferroni correction adjusted the threshold p-value to 0.00012. Yellow marking: assumptions for parametric testing were not met, non-parametric Spearman rank correlation analysis was performed.

| GD 108 | | | | | | | | |
| --- | --- | --- | --- | --- | --- | --- | --- | --- |
|  | Phenacetin  (human CYP1A2) | | Midazolam (human CYP3A4) | | Tolbutamide (human CYP2C9) | | Dextromethorphan (human CYP2D6) | |
|  | Pearson correlation coefficient | p-value | Pearson correlation coefficient | p-value | Pearson correlation coefficient | p-value | Pearson correlation coefficient | p-value |
| CYP1A2 | 0.4389 | 0.3839 | 0.3333 | 0.5186 | 0.7008 | 0.1209 | 0.4154 | 0.4128 |
| CYP2A19 | 0.2371 | 0.6511 | -0.7588 | 0.0803 | 0.4024 | 0.4289 | 0.0865 | 0.8706 |
| CYP2C33 | -0.1593 | 0.7631 | -0.776 | 0.0696 | 0.0248 | 0.9627 | 0.1879 | 0.7215 |
| CYP2C33v3 | 0.6456 | 0.1662 | -0.1644 | 0.7556 | 0.856 | 0.0296 | 0.7986 | 0.0568 |
| CYP2C34 (Fragment) | 0.8593 | 0.0283 | 0.5954 | 0.2124 | 0.8613 | 0.0275 | 0.4158 | 0.4123 |
| CYP2C36 | 0.7174 | 0.1085 | 0.8224 | 0.0445 | 0.4882 | 0.3259 | 0.3795 | 0.4581 |
| CYP2D6 (Fragment) | 0.1993 | 0.705 | -0.2973 | 0.5671 | -0.0798 | 0.8805 | 0.3548 | 0.4901 |
| CYP2D25 | 0.2223 | 0.6721 | 0.2096 | 0.6903 | 0.0745 | 0.8884 | 0.7658 | 0.0759 |
| CYP2E1_2 | 0.5145 | 0.2963 | -0.3035 | 0.5587 | 0.4099 | 0.4196 | 0.2853 | 0.5837 |
| CYP3A22 | 0.2 | 0.704 | -0.6 | 0.208 | 0.4286 | 0.3965 | -0.0857 | 0.8717 |
| CYP3A29 | -0.7564 | 0.0818 | 0.1373 | 0.7953 | -0.8955 | 0.0158 | -0.5526 | 0.2555 |
| CYP3A46 | 0.0446 | 0.9331 | -0.8911 | 0.0171 | 0.0708 | 0.894 | 0.2329 | 0.6569 |
| CYP4A21 | -0.5475 | 0.2609 | -0.2119 | 0.6869 | -0.25 | 0.6328 | -0.0488 | 0.9269 |
| CYP4A24 | -0.8253 | 0.0431 | -0.5842 | 0.2234 | -0.5404 | 0.2683 | -0.4149 | 0.4134 |
| CYP4V2_2a (Fragment) | 0.4212 | 0.4055 | 0.2997 | 0.5639 | 0.3093 | 0.5509 | 0.3134 | 0.5453 |
| CYP20A1 | -0.0286 | 0.9572 | -0.2571 | 0.6228 | 0.1429 | 0.7872 | -0.1429 | 0.7872 |
| CYP27A1 (Fragment) | -0.0827 | 0.8763 | -0.3015 | 0.5614 | 0.0523 | 0.9216 | -0.6959 | 0.1247 |
| CYP51A1 | -0.4857 | 0.3287 | -0.3714 | 0.4685 | -0.4286 | 0.3965 | -0.0286 | 0.9572 |

**Supplementary Table S4.2**: Pearson correlation analysis between CYP enzyme activity and protein abundance in liver microsomes originating from Göttingen Minipigs at PND 1. The analysis was performed for each CYP individually. The Bonferroni correction adjusted the threshold p-value to 0.00012.

| PND 1 | | | | | | | | | |
| --- | --- | --- | --- | --- | --- | --- | --- | --- | --- |
|  | Acetaminophen  (human CYP1A2) | | | Midazolam (human CYP3A4) | | Tolbutamide (human CYP2C9) | | Dextromethorphan (human CYP2D6) | |
|  | Pearson correlation coefficient | p-value | | Pearson correlation coefficient | p-value | Pearson correlation coefficient | p-value | Pearson correlation coefficient | p-value |
| CYP1A2 | 0.0054 | | 0.9899 | 0.1158 | 0.7848 | 0.1509 | 0.7213 | -0.0842 | 0.8428 |
| CYP2A19 | -0.369 | | 0.3683 | 0.06 | 0.8877 | -0.0769 | 0.8564 | -0.1216 | 0.7742 |
| CYP2C33 | 0.4796 | | 0.2291 | 0.6201 | 0.101 | 0.7377 | 0.0367 | 0.6845 | 0.0611 |
| CYP2C33v3 | 0.5942 | | 0.1204 | 0.1301 | 0.7588 | -0.1492 | 0.7244 | 0.6115 | 0.1072 |
| CYP2C34 (Fragment) | 0.504 | | 0.2029 | 0.5569 | 0.1516 | 0.3836 | 0.3482 | 0.619 | 0.1017 |
| CYP2C36 | 0.5208 | | 0.1857 | 0.2027 | 0.6303 | -0.051 | 0.9045 | 0.4908 | 0.2169 |
| CYP2D6 (Fragment) | 0.3352 | | 0.417 | -0.3281 | 0.4276 | -0.0788 | 0.8529 | 0.0091 | 0.983 |
| CYP2D25 | 0.5218 | | 0.1847 | 0.3469 | 0.3998 | 0.2894 | 0.4869 | 0.1272 | 0.7641 |
| CYP2E1_2 | 0.5909 | | 0.123 | 0.9218 | 0.0011 | 0.5651 | 0.1444 | 0.699 | 0.0537 |
| CYP3A22 | -0.2232 | | 0.5952 | 0.447 | 0.2669 | 0.264 | 0.5275 | -0.3085 | 0.4573 |
| CYP3A29 | 0.686 | | 0.0603 | 0.3597 | 0.3814 | 0.2269 | 0.5889 | 0.807 | 0.0155 |
| CYP3A46 | 0.1871 | | 0.6572 | -0.2291 | 0.5852 | -0.4538 | 0.2587 | 0.0892 | 0.8336 |
| CYP4A21 | -0.025 | | 0.9532 | 0.5194 | 0.1871 | 0.7167 | 0.0455 | 0.2013 | 0.6327 |
| CYP4A24 | - | | - | - | - | - | - | - | - |
| CYP4V2_2a (Fragment) | 0.2764 | | 0.5076 | 0.3184 | 0.4421 | 0.7706 | 0.0252 | 0.2621 | 0.5306 |
| CYP20A1 | 0.0042 | | 0.9921 | 0.377 | 0.3572 | 0.6293 | 0.0946 | 0.1752 | 0.6781 |
| CYP27A1 (Fragment) | -0.1474 | | 0.7276 | 0.2492 | 0.5517 | -0.0631 | 0.882 | 0.1031 | 0.8081 |
| CYP51A1 | 0.0329 | | 0.9383 | 0.0617 | 0.8847 | 0.1826 | 0.6652 | 0.4262 | 0.2924 |

**Supplementary Table S4.3**: Pearson correlation analysis between CYP enzyme activity and protein abundance in liver microsomes originating from Göttingen Minipigs at PND 3. The analysis was performed for each CYP individually. The Bonferroni correction adjusted the threshold p-value to 0.00012. Yellow marking: assumptions for parametric testing were not met, non-parametric Spearman rank correlation analysis was performed.

| PND 3 | | | | | | | | |
| --- | --- | --- | --- | --- | --- | --- | --- | --- |
|  | Acetaminophen  (human CYP1A2) | | Midazolam (human CYP3A4) | | Tolbutamide (human CYP2C9) | | Dextromethorphan (human CYP2D6) | |
|  | Pearson correlation coefficient | p-value | Pearson correlation coefficient | p-value | Pearson correlation coefficient | p-value | Pearson correlation coefficient | p-value |
| CYP1A2 | 0.0434 | 0.9187 | -0.2338 | 0.5773 | -0.4363 | 0.2798 | -0.4883 | 0.2195 |
| CYP2A19 | -0.0883 | 0.8352 | -0.1716 | 0.6844 | -0.4873 | 0.2206 | -0.3236 | 0.4342 |
| CYP2C33 | 0.0071 | 0.9867 | 0.0977 | 0.818 | -0.1166 | 0.7834 | 0.0957 | 0.8217 |
| CYP2C33v3 | 0.2143 | 0.6103 | 0.3333 | 0.4198 | -0.1905 | 0.6514 | -0.381 | 0.3518 |
| CYP2C34 (Fragment) | 0.1122 | 0.7914 | 0.068 | 0.873 | 0.5771 | 0.1342 | 0.4492 | 0.2642 |
| CYP2C36 | 0.1211 | 0.7752 | 0.037 | 0.9307 | 0.7295 | 0.04 | 0.4915 | 0.2161 |
| CYP2D6 (Fragment) | 0.1826 | 0.6652 | 0.1538 | 0.7162 | 0.2522 | 0.5468 | 0.3899 | 0.3397 |
| CYP2D25 | 0.291 | 0.4844 | 0.102 | 0.81 | 0.0182 | 0.966 | 0.1487 | 0.7252 |
| CYP2E1_2 | -0.2401 | 0.5668 | 0.029 | 0.9457 | -0.579 | 0.1326 | -0.4997 | 0.2073 |
| CYP3A22 | -0.0745 | 0.8608 | 0.3904 | 0.339 | 0.1895 | 0.6531 | 0.4726 | 0.237 |
| CYP3A29 | -0.1486 | 0.7255 | -0.1521 | 0.7192 | 0.3461 | 0.401 | 0.3003 | 0.4699 |
| CYP3A46 | 0.2232 | 0.5952 | 0.2851 | 0.4937 | 0.2296 | 0.5844 | 0.2713 | 0.5156 |
| CYP4A21 | -0.9021 | 0.0022 | -0.6315 | 0.093 | -0.7318 | 0.039 | -0.5551 | 0.1532 |
| CYP4A24 | - | - | - | - | - | - | - | - |
| CYP4V2_2a (Fragment) | -0.0678 | 0.8732 | -0.4449 | 0.2694 | -0.1581 | 0.7084 | -0.271 | 0.5162 |
| CYP20A1 | 0.2239 | 0.594 | -0.2852 | 0.4935 | -0.0198 | 0.963 | -0.1032 | 0.8078 |
| CYP27A1 (Fragment) | -0.3418 | 0.4073 | -0.1218 | 0.7738 | -0.1661 | 0.6943 | 0.0195 | 0.9634 |
| CYP51A1 | -0.3934 | 0.335 | -0.2499 | 0.5506 | -0.4578 | 0.254 | -0.4285 | 0.2895 |

**Supplementary Table S4.4**: Pearson correlation analysis between CYP enzyme activity and protein abundance in liver microsomes originating from Göttingen Minipigs at PND 7. The analysis was performed for each CYP individually. The Bonferroni correction adjusted the threshold p-value to 0.00012.

| PND 7 | | | | | | | | |
| --- | --- | --- | --- | --- | --- | --- | --- | --- |
|  | Acetaminophen  (human CYP1A2) | | Midazolam (human CYP3A4) | | Tolbutamide (human CYP2C9) | | Dextromethorphan (human CYP2D6) | |
|  | Pearson correlation coefficient | p-value | Pearson correlation coefficient | p-value | Pearson correlation coefficient | p-value | Pearson correlation coefficient | p-value |
| CYP1A2 | 0.645 | 0.0842 | 0.7404 | 0.0357 | 0.4977 | 0.2095 | 0.7506 | 0.0319 |
| CYP2A19 | 0.2414 | 0.5647 | 0.6289 | 0.0948 | 0.4018 | 0.3238 | 0.5315 | 0.1752 |
| CYP2C33 | 0.3237 | 0.4341 | 0.4675 | 0.2428 | 0.7371 | 0.0369 | 0.3444 | 0.4035 |
| CYP2C33v3 | 0.4658 | 0.2447 | 0.5146 | 0.192 | -0.4479 | 0.2658 | -0.0444 | 0.9168 |
| CYP2C34 (Fragment) | -0.0474 | 0.9112 | -0.2791 | 0.5032 | 0.4367 | 0.2793 | -0.1146 | 0.787 |
| CYP2C36 | -0.1513 | 0.7206 | -0.1685 | 0.6899 | 0.5704 | 0.1399 | 0.0727 | 0.8642 |
| CYP2D6 (Fragment) | 0.191 | 0.6505 | 0.377 | 0.3573 | 0.6272 | 0.096 | 0.9264 | 0.0009 |
| CYP2D25 | 0.2575 | 0.5381 | 0.6866 | 0.06 | 0.7665 | 0.0265 | 0.9349 | 0.0007 |
| CYP2E1_2 | 0.2794 | 0.5027 | -0.0861 | 0.8393 | 0.2674 | 0.522 | 0.0171 | 0.9679 |
| CYP3A22 | 0.3691 | 0.3682 | 0.9633 | 0.0001 | 0.4132 | 0.3089 | 0.646 | 0.0835 |
| CYP3A29 | 0.1736 | 0.681 | 0.8737 | 0.0046 | 0.4634 | 0.2475 | 0.5632 | 0.146 |
| CYP3A46 | 0.1713 | 0.685 | -0.0033 | 0.9938 | 0.4779 | 0.2311 | 0.7079 | 0.0486 |
| CYP4A21 | 0.2306 | 0.5827 | 0.4014 | 0.3243 | 0.0424 | 0.9205 | 0.3615 | 0.3789 |
| CYP4A24 | 0.2919 | 0.7081 | -0.3468 | 0.6532 | -0.6373 | 0.3627 | -0.5363 | 0.4637 |
| CYP4V2_2a (Fragment) | -0.6894 | 0.0586 | -0.4123 | 0.3101 | 0.1397 | 0.7415 | 0.1396 | 0.7416 |
| CYP20A1 | 0.5538 | 0.1544 | 0.4672 | 0.2431 | 0.7414 | 0.0353 | 0.7324 | 0.0388 |
| CYP27A1 (Fragment) | 0.8713 | 0.0048 | 0.1574 | 0.7098 | 0.1069 | 0.8011 | 0.027 | 0.9494 |
| CYP51A1 | -0.0193 | 0.9639 | -0.6786 | 0.0643 | -0.065 | 0.8784 | -0.4985 | 0.2086 |

**Supplementary Table S4.5**: Pearson correlation analysis between CYP enzyme activity and protein abundance in liver microsomes originating from Göttingen Minipigs at PND 28. The analysis was performed for each CYP individually. The Bonferroni correction adjusted the threshold p-value to 0.00012.

| PND 28 | | | | | | | | |
| --- | --- | --- | --- | --- | --- | --- | --- | --- |
|  | Acetaminophen  (human CYP1A2) | | Midazolam (human CYP3A4) | | Tolbutamide (human CYP2C9) | | Dextromethorphan (human CYP2D6) | |
|  | Pearson correlation coefficient | p-value | Pearson correlation coefficient | p-value | Pearson correlation coefficient | p-value | Pearson correlation coefficient | p-value |
| CYP1A2 | 0.543 | 0.1643 | -0.3066 | 0.4601 | -0.3784 | 0.3553 | 0.3945 | 0.3335 |
| CYP2A19 | 0.3248 | 0.4325 | -0.1216 | 0.7743 | -0.24 | 0.5669 | 0.1885 | 0.6548 |
| CYP2C33 | 0.0719 | 0.8657 | -0.0432 | 0.9192 | -0.0469 | 0.9121 | 0.3228 | 0.4354 |
| CYP2C33v3 | -0.3976 | 0.3294 | 0.3682 | 0.3695 | -0.2069 | 0.6213 | -0.2393 | 0.5681 |
| CYP2C34 (Fragment) | 0.094 | 0.8247 | 0.1353 | 0.7494 | 0.1531 | 0.7175 | 0.3844 | 0.347 |
| CYP2C36 | -0.6337 | 0.0916 | 0.1251 | 0.7678 | 0.3849 | 0.3464 | 0.0689 | 0.8713 |
| CYP2D6 (Fragment) | 0.0236 | 0.9558 | -0.0583 | 0.8909 | 0.0193 | 0.9638 | 0.4534 | 0.2592 |
| CYP2D25 | 0.0752 | 0.8595 | 0.0481 | 0.91 | -0.1209 | 0.7755 | 0.3474 | 0.3992 |
| CYP2E1_2 | 0.581 | 0.1309 | 0.0285 | 0.9465 | -0.0944 | 0.824 | 0.0403 | 0.9246 |
| CYP3A22 | 0.0463 | 0.9133 | 0.9114 | 0.0016 | 0.4164 | 0.3048 | -0.0504 | 0.9056 |
| CYP3A29 | -0.4032 | 0.322 | 0.2564 | 0.5399 | 0.324 | 0.4337 | 0.1669 | 0.6929 |
| CYP3A46 | -0.5439 | 0.1635 | 0.4679 | 0.2423 | 0.281 | 0.5002 | 0.1389 | 0.7428 |
| CYP4A21 | -0.308 | 0.458 | 0.2094 | 0.6187 | -0.0581 | 0.8914 | 0.3864 | 0.3444 |
| CYP4A24 | -0.5676 | 0.6157 | -0.068 | 0.9567 | -0.4778 | 0.6828 | 0.4504 | 0.7025 |
| CYP4V2_2a (Fragment) | -0.2012 | 0.6327 | -0.3926 | 0.336 | -0.4113 | 0.3113 | -0.1125 | 0.7909 |
| CYP20A1 | -0.2012 | 0.6327 | -0.3926 | 0.336 | -0.4113 | 0.3113 | -0.1125 | 0.7909 |
| CYP27A1 (Fragment) | -0.4681 | 0.2421 | -0.6128 | 0.1062 | -0.5275 | 0.1791 | -0.5305 | 0.1762 |
| CYP51A1 | -0.1947 | 0.644 | 0.2567 | 0.5393 | -0.1238 | 0.7703 | -0.1844 | 0.662 |

**Supplementary Table S4.6**: Pearson correlation analysis between CYP enzyme activity and protein abundance in liver microsomes originating from adult male Göttingen Minipigs. The analysis was performed for each CYP individually. The Bonferroni correction adjusted the threshold p-value to 0.00012.

| Adult male | | | | | | | | |
| --- | --- | --- | --- | --- | --- | --- | --- | --- |
|  | Acetaminophen  (human CYP1A2) | | Midazolam (human CYP3A4) | | Tolbutamide (human CYP2C9) | | Dextromethorphan (human CYP2D6) | |
|  | Pearson correlation coefficient | p-value | Pearson correlation coefficient | p-value | Pearson correlation coefficient | p-value | Pearson correlation coefficient | p-value |
| CYP1A2 | 0.8291 | 0.1709 | 0.1569 | 0.8431 | -0.1067 | 0.8933 | 0.7025 | 0.2975 |
| CYP2A19 | 0.9513 | 0.0487 | 0.4377 | 0.5623 | 0.4967 | 0.5033 | 0.9812 | 0.0188 |
| CYP2C33 | -0.7831 | 0.2169 | -0.1096 | 0.8904 | 0.1778 | 0.8222 | -0.6436 | 0.3564 |
| CYP2C33v3 | -0.3702 | 0.6298 | 0.0342 | 0.9658 | 0.4976 | 0.5024 | -0.1633 | 0.8367 |
| CYP2C34 (Fragment) | -0.8703 | 0.1297 | -0.8883 | 0.1117 | -0.6976 | 0.3024 | -0.8585 | 0.1415 |
| CYP2C36 | -0.1703 | 0.8297 | 0.7075 | 0.2925 | 0.7137 | 0.2863 | -0.0747 | 0.9253 |
| CYP2D6 (Fragment) | 0.9794 | 0.0206 | 0.3907 | 0.6093 | 0.3392 | 0.6608 | 0.9581 | 0.0419 |
| CYP2D25 | 0.7878 | 0.2122 | 0.2417 | 0.7583 | 0.4755 | 0.5435 | 0.8718 | 0.1282 |
| CYP2E1_2 | 0.7605 | 0.2395 | -0.0703 | 0.9297 | -0.2152 | 0.7848 | 0.6574 | 0.3426 |
| CYP3A22 | 0.4236 | 0.5764 | 0.987 | 0.013 | 0.8352 | 0.1648 | 0.4608 | 0.5392 |
| CYP3A29 | -0.6703 | 0.3297 | -0.2272 | 0.7728 | -0.5463 | 0.4537 | -0.7922 | 0.2078 |
| CYP3A46 | -0.5283 | 0.4717 | 0.2988 | 0.7012 | 0.5129 | 0.4871 | -0.3815 | 0.6185 |
| CYP4A21 | -0.5989 | 0.4011 | -0.0353 | 0.9647 | 0.3581 | 0.6419 | -0.4171 | 0.5829 |
| CYP4A24 | -0.7825 | 0.2175 | -0.9493 | 0.0507 | -0.85 | 0.15 | -0.8188 | 0.1812 |
| CYP4V2_2a (Fragment) | 0.148 | 0.852 | -0.1245 | 0.8755 | 0.3787 | 0.6213 | 0.3265 | 0.6735 |
| CYP20A1 | 0.8156 | 0.1844 | 0.2647 | 0.7353 | 0.4758 | 0.5242 | 0.8919 | 0.1081 |
| CYP27A1 (Fragment) | -0.7382 | 0.2618 | -0.5134 | 0.4866 | -0.7658 | 0.2342 | -0.8646 | 0.1354 |
| CYP51A1 | -0.0959 | 0.9041 | 0.1662 | 0.8338 | 0.6435 | 0.3565 | 0.119 | 0.881 |
